# Signal enhancement of lipids and glycans using ammonium fluoride for IR-MALDESI mass spectrometry imaging

**DOI:** 10.1007/s00216-025-06197-0

**Published:** 2025-11-18

**Authors:** Seth M. Eisenberg, Alora R. Dunnavant, Tana V. Palomino, David C. Muddiman

**Affiliations:** Biological Imaging Laboratory for Disease and Exposure Research (BILDER), Department of Chemistry, North Carolina State University, Raleigh, NC 27695 USA

**Keywords:** IR-MALDESI, Mass spectrometry imaging, Electrospray doping, Ammonium fluoride, Glycans

## Abstract

Mass spectrometry imaging (MSI) is an analytical technique that enables the simultaneous detection and localization of hundreds to thousands of molecules across a tissue surface. The addition of ammonium fluoride to the electrospray or to tissue samples has been shown to increase the signal of lipids in both nano-DESI-MSI and MALDI-MSI, respectively. Herein, this strategy is applied to infrared matrix-assisted laser desorption electrospray ionization (IR-MALDESI) which demonstrated signal enhancement for both lipids and *N*-linked glycans using 70 µM and 350 µM NH_4_F in 50% ACN with 1 mM acetic acid as the ESI solvent, respectively. Up to a 93-fold increase in signal was observed for these biomolecules without any change in sample preparation or to the mass spectrometer parameters, including detecting lipids not seen with the control ESI solvent. On average, lipids were enhanced by eightfold, and the glycan signal increased by 1.2-fold. The enhancement factor for lipids was significantly impacted by lipid class, while glycan enhancement was affected by charge state and the observed adduct.

**Graphical Abstract:**

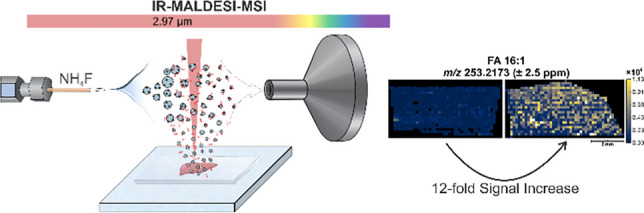

**Supplementary Information:**

The online version contains supplementary material available at 10.1007/s00216-025-06197-0.

## Introduction

Mass spectrometry imaging (MSI) is a unique application of mass spectrometry, wherein ions are both detected and localized spatially, creating a 2D or 3D map of the location and abundance of thousands of detected analytes [1]. Using infrared matrix-assisted laser desorption electrospray ionization (IR-MALDESI), a hybrid technique between matrix-assisted laser desorption/ionization (MALDI) and electrospray (ESI), a range of biomolecules can be ionized and measured [2]. In nearly all cases of mass spectrometry analysis, an increased signal-to-noise ratio is desirable, especially when operating at atmospheric pressure, where background signal can confound spectra and cause ionization suppression of biologically relevant analytes [3]. ESI doping involves the addition of various metals or salts to an electrospray for mass spectrometry detection to either (1) increase ion signal or (2) obtain structural information. In IR-MALDESI, silver nitrate has previously been used as an ESI dopant to increase the ion abundance of unsaturated lipids [4]. Lithium has been used with tandem mass spectrometry (MS/MS) to induce cross-ring fragmentation of glycans, enabling increased characterization and identification of isomers [5].

Ammonium fluoride has been used as an ESI dopant in negative ion mode in many applications, ranging from LC–MS [6, 7] to MSI. In nano-DESI-MSI, a 10 to 110-fold increase in lipid signals was observed when 500 µM NH_4_F was included in the nano-DESI solvent [8]. When 500 µM NH_4_F was added as a co-matrix for MALDI-MSI, lipid signals were improved threefold [9]. In LC–MS, the addition of 200 µM NH_4_F to the LC solvent system resulted in a 2 to 22-fold improvement in sensitivity in detecting a variety of small pharmaceutical compounds [10]. Based on the work presented here, it has also been utilized for quantitative MSI to improve the limit of detection and measurement consistency of glutathione [11].

Ammonium fluoride is hypothesized to improve ion abundance via the highly electronegative fluoride ion abstracting a proton(s) from a neutral analyte [7], thereby forming [M-H^+^]^−^. While targeted mass spectrometry can yield quantification of known, pre-selected targets, this prevents the detection of other biomolecules. Performing an untargeted assay can result in the detection of all the lipids, metabolites, and other biomolecules present in a system, and can provide information about how they interact and co-localize. Thus, methodologies such as ESI doping that can increase the ion abundance of all biomolecules are extremely valuable as they are more widely applicable to any research focus.

 Herein, we demonstrate the use of ammonium fluoride as an ESI dopant for IR-MALDESI. A range of different molecules from lipids and glycans is shown to increase abundance up to 93-fold, with an average abundance increase of eight-fold for lipids, four-fold for glycans from a tissue extract, and 1.2-fold for glycans on tissue. Nearly all analytes showed an increase in ion abundance, the extent of which was dependent on analyte acidity, lipid class, charge state, and adduct.

## Experimental

### Materials

LC–MS grade water, acetonitrile (ACN), and acetic acid were purchased from Thermo Fisher Scientific (Nazareth, PA, USA). Ammonium fluoride (> 98.0%) was purchased from Sigma Aldrich (St. Louis, MO, USA). Splashmix lipid mixture was purchased from Avanti (Cat #330707, Birmingham, AL, USA). Nitrogen gas was purchased from Arc3 gases (Raleigh, NC, USA).

### Sample preparation

The mouse liver was obtained from the Ghashghaei laboratory at the North Carolina State University (NCSU) College of Veterinary Medicine where the animals were cared for in accordance with the NCSU Institutional Animal Care and Use Committee (IACUC). Once sacrificed, the liver was harvested, flash frozen on dry ice, and stored at −80 °C until sectioning to a thickness of 7 µm using a Leica CM1950 cryostat (Buffalo Grove, IL, USA) at −15 °C and thaw mounting onto a plain glass microscope slide (1 mm thickness) [12].

*N*-linked glycans were prepared from bovine fetuin (Sigma Aldrich) as previously described [13]. Briefly, a mixture of 250 µg of bovine fetuin was loaded onto a 10 kDa molecular weight cut-off filter (MWCO) with dithiothreitol (Sigma Aldrich) and 100 mM ammonium bicarbonate (Sigma Aldrich). The denatured proteins were alkylated with iodoacetamide (Sigma Aldrich). One thousand units of PNGase F PRIME-LY (Bulldog Bio, NJ, USA) were added to the filter to cleave the *N*-linked glycans, followed by overnight incubation at 37 °C. Glycans were eluted and dried to completion in a vacuum desiccator and resuspended in LC–MS grade water.

The formalin-fixed paraffin-embedded (FFPE) human kidney samples were obtained from the Drake laboratory at the Medical University of South Carolina. The kidney was sectioned to 3 µm using an HM-355S rotary microtome (Epredia, Kalamazoo, MI, USA) and mounted onto uncharged slides. The slides were prepared for imaging as previously described [14]. Briefly, the wax was melted at 60 °C (1 h) before dewaxing with washes consisting of 2 × xylenes (3 min), 2 × 100% ethanol (1 min), 1 × 95% ethanol (1 min), 1 × 70% ethanol (1 min), and 2 × 100% LC–MS grade water (3 min). The slides were vacuum dried and treated to antigen retrieval, placing the slides in a mailer filled with citraconic acid buffer and heating in a vegetable steamer. A buffer exchange was performed, slowly replacing the buffer with LC–MS grade water. After drying, 100 µg/mL of PNGase F PRIME-LY was prepared and sprayed onto the slides using a TM-Sprayer (HTX Imaging, Carrboro, NC, USA). The slides were incubated in a humidity chamber (2 h), with a relative humidity reaching 95%, and placed at −80 °C before IR-MALDESI analysis.

### IR-MALDESI-MSI analysis

**Fig. 1:**
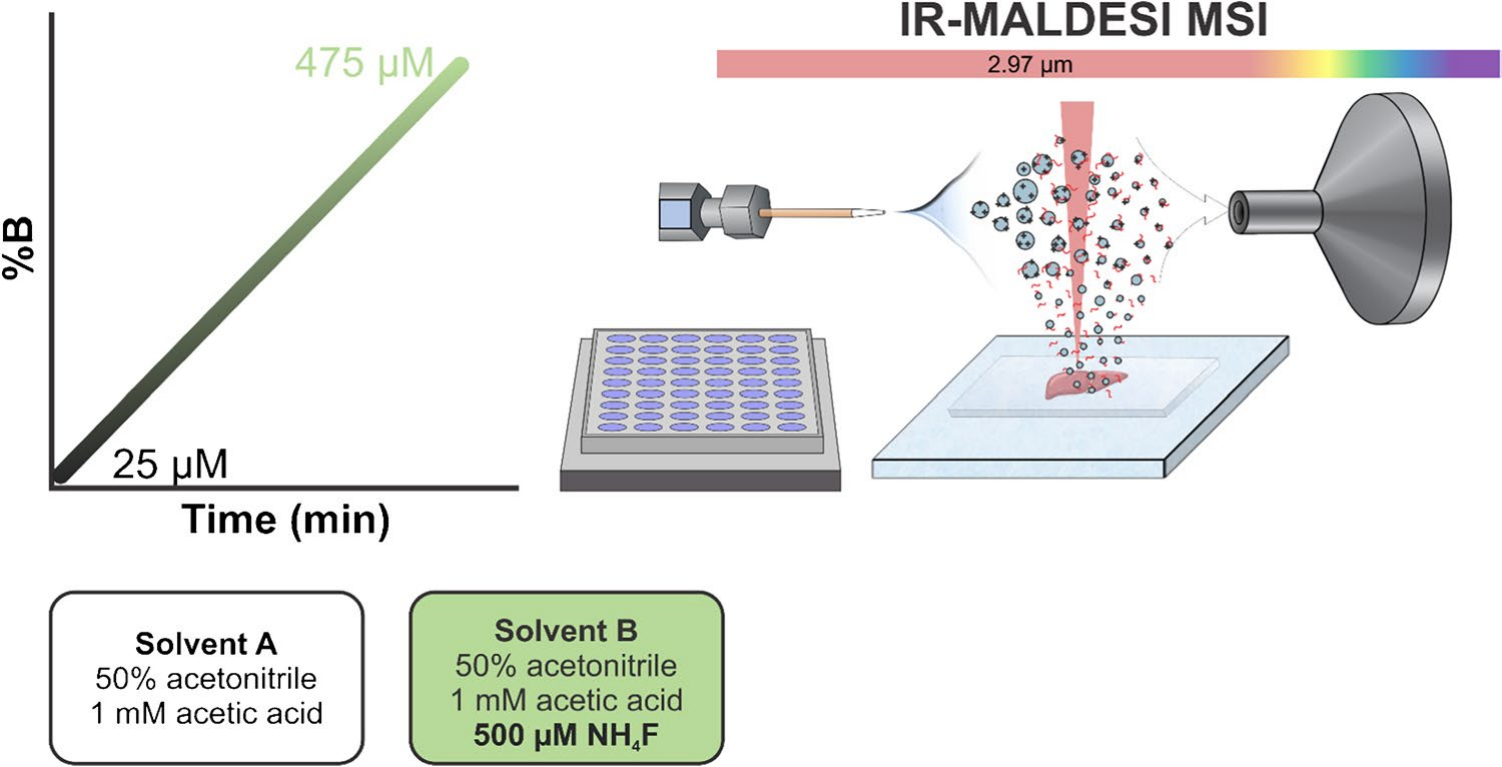
Methodology to determine the ideal NH_4_F concentration for IR-MALDESI MSI. A nano LC (Solvent A: 50% ACN, 1 mM acetic acid; Solvent B: 50% ACN, 1 mM acetic acid, 500 µM NH_4_F) was used with a gradient increasing from 5 to 95% B. This LC gradient was used as the electrospray solvent for IR-MALDESI-MSI of Splashmix (well plate) and of mouse liver (mounted slide), resulting in an optimized NH_4_F concentration based on the peak ion abundance of detected lipids

The next-generation IR-MALDESI source [15] was interfaced with an Orbitrap Exploris 240 mass spectrometer (Thermo Fisher Scientific, Bremen, Germany), as was previously described [2]. For tissue imaging, the slide-mounted sample was placed on a Peltier-cooled stage inside a humidity-controlled enclosure. The enclosure was purged with inert nitrogen gas to reach <12% relative humidity (RH) before cooling the stage to −10 °C. After tissue cooling and temperature stabilization, the nitrogen flow was stopped and the enclosure was opened to allow an increase in RH, thereby depositing a thin ice layer on the tissue. After ice deposition, the enclosure was shut and purged with nitrogen to prevent further ice growth. A mid-IR laser (JGM Associates, Inc., Burlington, MA, USA) was fired at 2940 nm at 6 ppb, with 2.1 mJ/burst reaching the tissue to resonantly excite the O–H stretching vibrational mode of the endogenous water within the tissue and the exogenously applied ice matrix. For Splashmix and bovine fetuin glycan analysis, a well plate was positioned, and the solution was ablated by the mid-IR laser without the use of an ice matrix. In either case, the laser shot resulted in the ablation of neutral molecules, which partition into the orthogonal electrospray plume before being ionized in an electrospray-like mechanism. The ESI solvent consisted of 50% ACN, 1 mM acetic acid (Solvent A) and was flowed at 1.2 µL/min with an applied voltage of 3.6 kV for all experiments. An EASY nanoLC pump (Thermo Fisher Scientific) with a gradient was employed to optimize the NH_4_F dopant concentration. Solvent A consisted of 50% ACN with 1 mM acetic acid while solvent B was 50% ACN, 1 mM acetic acid [16], and 500 µM of NH_4_F. A gradient was employed from 5 to 95% B, creating a range from 25 to 475 µM NH_4_F to measure the ion abundance of either Splashmix or liver tissue (Fig. 1).

To initially optimize the concentration of NH_4_F, the ion abundance at each NH_4_F concentration was compared to find the maximal lipid signal. Once the ideal concentration was determined, the extent of the signal increase for lipids and glycans was determined in mouse liver and human kidney, respectively. For Splashmix and mouse liver lipid imaging, data were collected in negative centroid mode at a resolving power of 240,000_FWHM_ at *m/z* 200 with the EASY-IC internal calibrant fluoranthene ([M^●+^] *m/z* 202.0777) enabled, using a 15 ms injection time. Mass spectra were collected from *m/z* 150 to *m/z* 1500 and the multi-injection ratio at 10. For glycan imaging, data were collected in negative profile mode, using a 90 ms injection time. Mass spectra were recorded from *m/z* 500 to *m/z* 2000 and the multi-injection ratio at 4. In all experiments, the automatic gain control function was disabled and the S-lens RF value was set at 70%.

### Data analysis

Raw mass spectra were analyzed in XCalibur (Thermo Fisher Scientific). The .RAW data files were also converted to .mzML using MS Convert by ProteoWizard [17] and then converted to .imzML [18] for analysis in MSiReader v3.2 (MSI Software Solutions, Raleigh, NC) [19, 20]. Further external mass correction was not needed, as ± 2-ppm mass measurement accuracy (MMA) was observed for all annotations. All ion images are shown with an associated SMART, a data reporting standard to provide more information about how an ion image was obtained. This acronym indicates the step size and laser spot size, molecular identification confidence, annotations, resolving power, and time of acquisition [21].

For evaluating the effect of NH_4_F on lipids, the imaged liver was putatively annotated using METASPACE [22], at a 10% FDR using the LIPIDMAPS database. All background annotations were manually excluded by comparison against a background ESI spectrum, generating a list of putatively annotated on-tissue lipids. The summed abundance of those lipids at each concentration of NH_4_F was compared to determine the maximal abundance, indicating which concentration led to the largest increase in signal. To evaluate the effect of NH_4_F on the ion abundance of glycans, the detected masses were uploaded to GlycoMod, a theoretical glycan database, providing *N*-linked glycan annotations within a 2.5 ppm mass tolerance. Glycan structures were confirmed using GlyConnect, a database which reports previously detected and published biological species. The summed glycan abundance at each NH_4_F concentration was compared to determine the concentration where glycan ion abundances were most improved.

## Results and discussion

### Effect of ammonium fluoride on lipid abundance

**Fig. 2:**
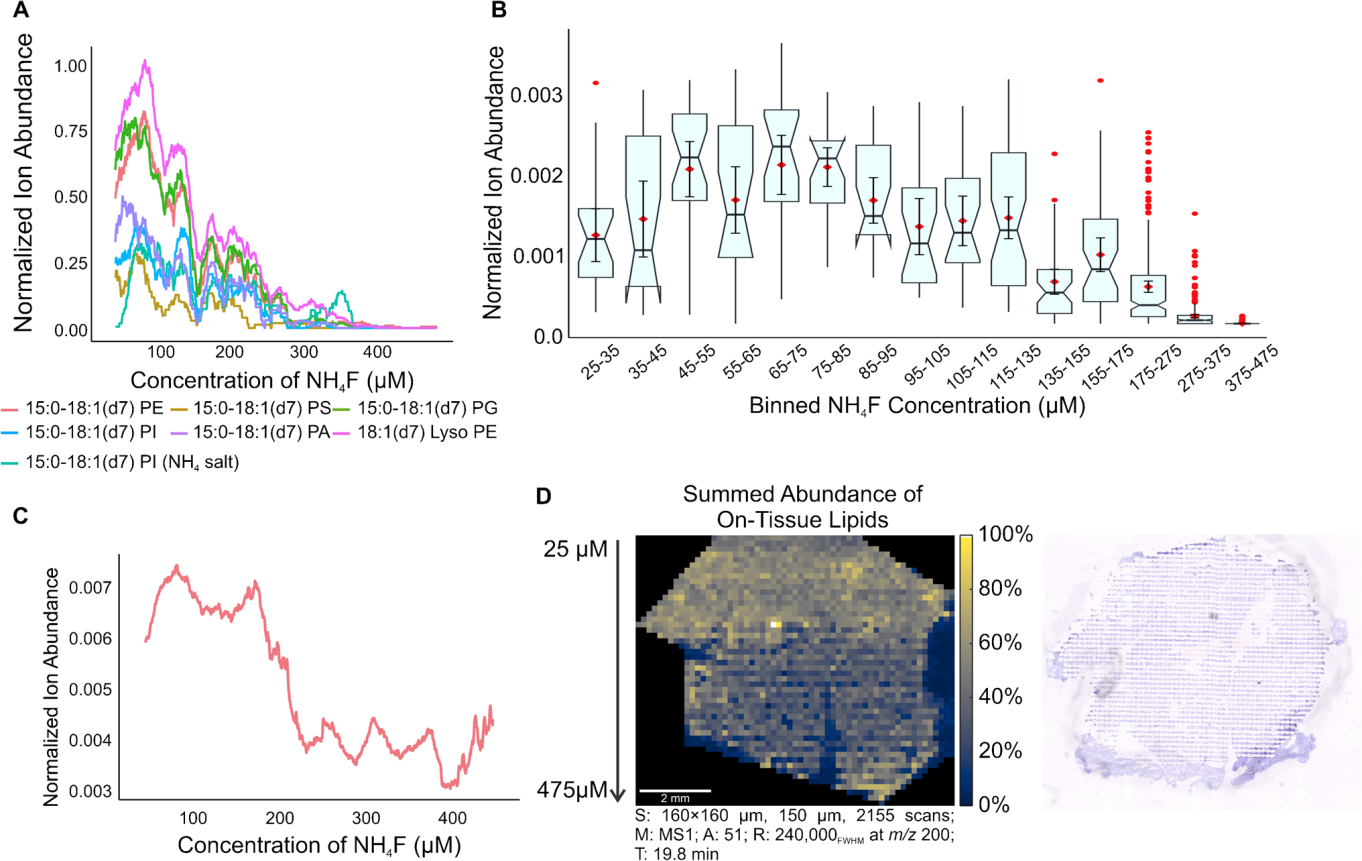
**A** Effect of NH_4_F concentration on ion abundance of 7 lipids in Avanti Splashmix. Abundance curves are smoothed using box car averaging, each box containing 50 points. **B** Notch box plot of binned NH_4_F concentration to determine the optimal concentration for signal enhancement, found at 70 µM. **C** Effect of NH_4_F concentration on lipid ion abundance of all on-tissue lipids observed in mouse liver. Abundance curves are smoothed using box car averaging, each box containing 100 points. The increased sample complexity showed minimal effect on the optimized concentration of NH_4_F, resulting in a peak at 70 µM. **D** Summed ion abundance heatmap of mouse liver with an applied NH_4_F gradient, showing higher abundance near the beginning of the gradient. The ions present in this heatmap represent all the lipids detected on tissue (51 lipids), normalized to the total ion chromatogram (TIC). An optical image of the tissue is also shown.

To establish an ideal concentration for NH_4_F, an Avanti Splashmix was analyzed using a continuous gradient from 25 to 475 µM over 10 min (Fig. 1). As the concentration of NH_4_F increased, the lipid signals increased until reaching ~70 µM NH_4_F, after which the lipid signal began to decrease (Fig. 2A and B). When the concentration of NH_4_F became too high, it likely began to increasingly ionize background ions, leading to ion suppression of the on-tissue lipids and increased noise.

To ensure increased sample complexity would not alter the optimized NH_4_F concentration, the gradient experiment was repeated while imaging liver tissue. Because the liver is a relatively homogenous sample, comparing the summed abundance of on-tissue ions at each concentration of ammonium fluoride allows for a direct comparison of the doping impact on ion abundance (Fig. 2C and D). This confirmed the concentration found using Splashmix, showing an ideal concentration ranging between 60 and 80 µM, with an optimal concentration at 70 µM. The 51 lipids summed in Fig. 2C and D are all detected on-tissue lipids, putatively annotated by METASPACE. The identifications can be seen in Supplemental Information (Table S1).

The Splashmix analyzed has a series of lipids with the same chain length and degree of unsaturation, allowing for an exploration of the effect of functional group (Fig. 2A). Specifically, PI and PS show the smallest signal enhancement, suggesting the large functional groups present may prevent deprotonation by F^−^ by steric hindrance. Meanwhile, the PA analyzed lies in the middle, showing moderate enhancement. The PG shows high signal enhancement, potentially caused by the multiple hydroxyl groups available for deprotonation. Surprisingly, PE shows very high signal enhancement by NH_4_F, unexpected because the amine group present would cause an increase in pKa and decrease the acidity of the available protons. However, this effect may be mitigated in a pure sample with little competition for ionization.

**Fig. 3:**
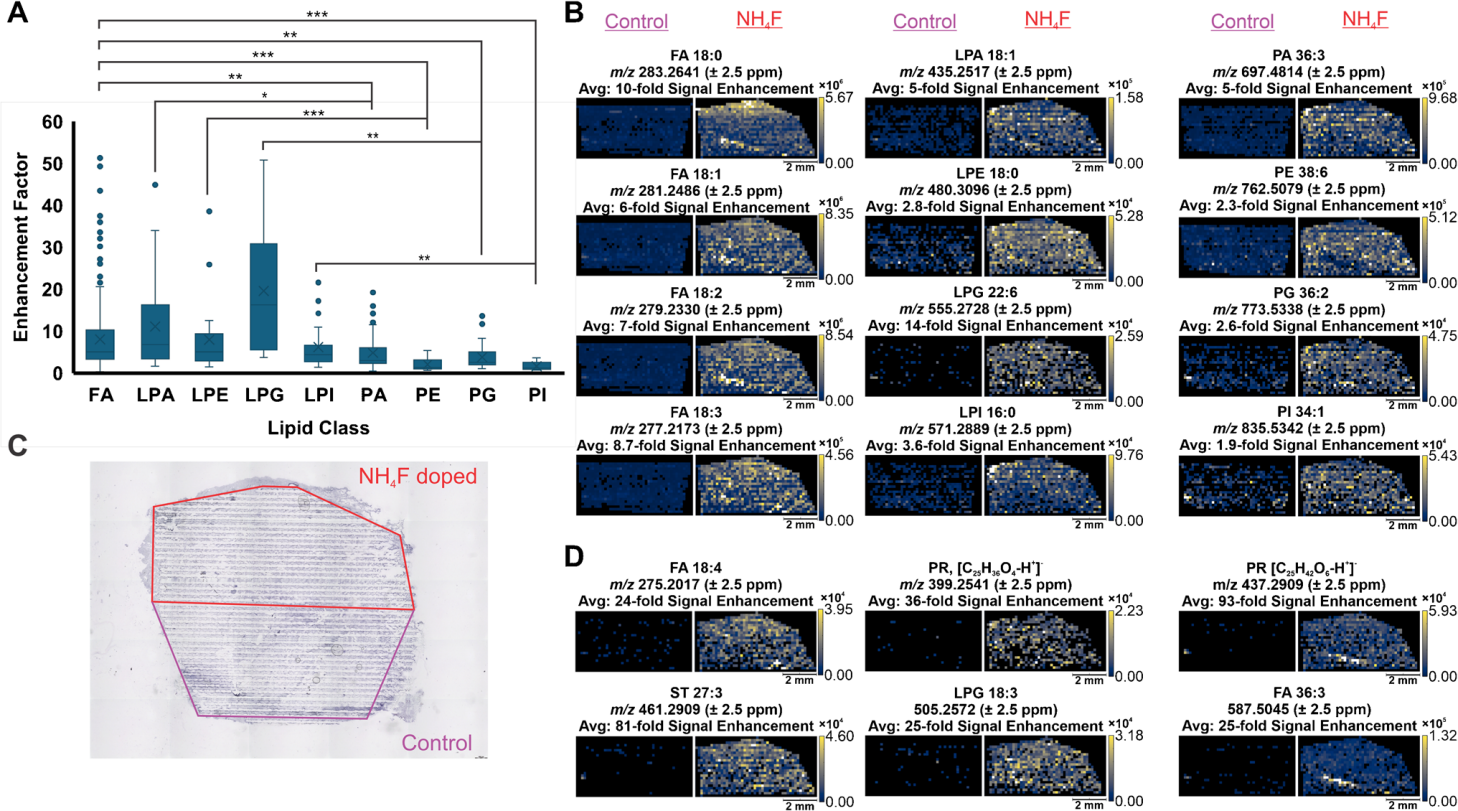
** A** Box and whiskers plot showing signal enhancement for all 182 lipids detected in tissue by lipid class. FAs showed significantly higher signal enhancement than PA, PE, PG, and PI lipids. Smaller lipids (LPA, LPE, LPG, LPI) showed significantly higher signal enhancement than larger counterparts (PA, PE, PG, PI). Statistical comparisons were performed with a Nemenyi test, *p*-value = 0.05, Tukey correction. *p-value < 0.05, **p-value < 0.01, ***p-value < 0.0001. **B** Ion heatmaps showing abundance of representative lipids for each class, demonstrating the increase in abundance from control to NH_4_F-doped. The putative identification for each lipid is provided, as well as the average signal enhancement across 4 replicate tissues. **C** Optical image of the liver shown in the ion heatmaps showing which half was imaged with each ESI condition. **D** Ion heatmaps of representative ions that were only detected when using NH_4_F, resulting in extremely high enhancement factors

To determine the effect and benefits of NH_4_F doping on tissue, MSI was performed by imaging one half of a tissue with 70 µM NH_4_F-doped ESI and the other half with control ESI. This method significantly reduces any biological variability and allows for a direct comparison of abundance change in the same tissue based on change in ESI composition. It was also shown that there was no meaningful increase in background noise associated with the increase in lipid signal (Supplemental Information, Fig. S2). In tissue, 182 m*/z* were detected and putatively identified by METASPACE as lipids, and the effect of doping on various lipid categories and classes was evaluated (Fig. 3A). The statistical differences between each lipid class were evaluated using a Nemenyi test with a Tukey correction and a p-value = 0.05. All p-values are available in Supplemental Information (Table 2).

On average, fatty acids (FAs) were enhanced by eightfold, while glycerophospholipids (GPs) showed a 5.5-fold increase, a statistically significant increase evaluated by a Kruskall-Wallis test. Likely, fatty acids experience a greater amount of deprotonation by F^−^ due to the decreased pKa from the presence of a carboxylic acid. Previous work has demonstrated a correlation between the signal enhancement, fatty acid chain length, and number of double bonds [8]. In IR-MALDESI, it was found that for fatty acids with the same number of double bonds, increasing chain length did not cause a significant change in signal enhancement when evaluated by a Kruskall-Wallis test with p-value = 0.05. Furthermore, it was observed that for fatty acids of the same chain length, increasing double bonds showed an increase in signal abundance; however, this increase was not statistically significant by Kruskall-Wallis with *p*-value = 0.05. This suggests that fatty acids are generally enhanced by NH_4_F equally across the *m/z* range, without regard for deprotonation or chain length (Fig. 3B). Ion heatmaps are shown as representative examples comparing the abundance seen across tissue halves, with and without NH_4_F, along with an optical image of the tissue shown (Fig. 3B, C).

When GPs are broken down into lipid classes, smaller GPs such as lysophosphatidic acid (LPA), lysophosphatidylethanolamine (LPE), lysylphosphatidylglycerol (LPG), and lysophosphatidylinositol (LPI) showed significantly higher enhancement factors than their larger counterparts phosphatidic acid (PA), phosphatidylethanolamine (PE), phosphatidylglycerol (PG), and phosphatidylinositol (PI) (Fig. 3A). These larger lipids may be harder to deprotonate due to steric hindrance from longer carbon chains. Considering these have similar functional groups, this lends credence to the hypothesis that steric hindrance can prevent the interaction of fluoride with hydrogen atoms. Interestingly, in contrast to what was observed in Splashmix, PE lipids showed very low signal enhancement, likely caused by their amine functional group, increasing the pKa of the lipid, making it more difficult to deprotonate. Similarly, PI showed low signal enhancement, matching what was seen in Splashmix, and further suggesting the inositol sugar is not easily deprotonated and can sterically hinder the lipid-fluoride interaction.

Lipids that showed signal enhancement factors greater than 20-fold typically were not detected in the control half in any significant manner (Fig. 3D). A total of 14 lipids were seen only with NH_4_F, although no clear trend indicating which lipids would be newly detected was observed (Supplemental Table 3). Additionally, only a few prenol lipids (PR) were detected, but they showed significant signal enhancement by NH_4_F doping.

When comparing these results with those observed by nano-DESI and MALDI, the optimized concentration was significantly lower than those identified for the other techniques. The signal enhancement observed was greater than for MALDI-MSI but showed similar improvements to those reported by nano-DESI, although the reported effects of increasing fatty acyl chain length and degree of unsaturation were not observed.

### Effect of NH_4_F on glycan abundance

IR-MALDESI is uniquely capable of detecting and analyzing glycans since it is both sensitive to negative mode ions and a soft ionization method, preventing the fragmentation of key highly labile monosaccharides such as sialic acid [23]. However, because glycans are detected with low abundance, their detection can be difficult, thus highly incentivizing the demonstration of increased ion abundance through the addition of a dopant, without requiring any modifications to the sample preparation or the mass spectrometer parameters.

**Fig. 4:**
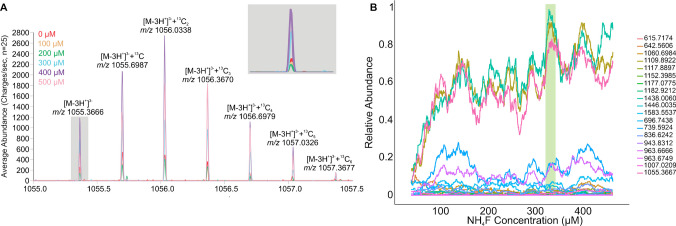
**A** Mass spectrum of glycan H5N4F1S1 showing the isotopic distribution at each concentration of NH_4_F (H = Hexose, N = N-Acetylglucosamine, F = Fucose, S = N-Acetylneuraminic acid). The signal of each peak increased with the concentration of NH_4_F until reaching a peak 400 µM. **B** Summed glycan abundance using a gradient from 0 to 500 µM NH_4_F, demonstrating an overall signal peak at 350 µM NH_4_F

To optimize the ammonium fluoride concentration needed to increase glycan signal and to account for their low abundance and heterogeneity in tissue, droplets of cleaved glycans from bovine fetuin were initially used. Glycans cleaved from bovine fetuin have been extensively studied by IR-MALDESI, and the previously identified glycans matched those observed in this experiment [24]. Contrasting significantly with what was observed for lipids, increasing the concentration of NH_4_F in ESI led to a significant increase in glycan abundance up to 350 µM (Fig. 4A and B). Overall, a four-fold increase in glycan signal from cleaved bovine fetuin *N*-glycans was observed with optimal NH_4_F concentration. This result, observed in liquid samples, can have a significant impact in the field of glycan profiling, commonly used in pharmaceutical production and often required for FDA approval, especially for monoclonal antibodies [25–27].

**Fig. 5:**
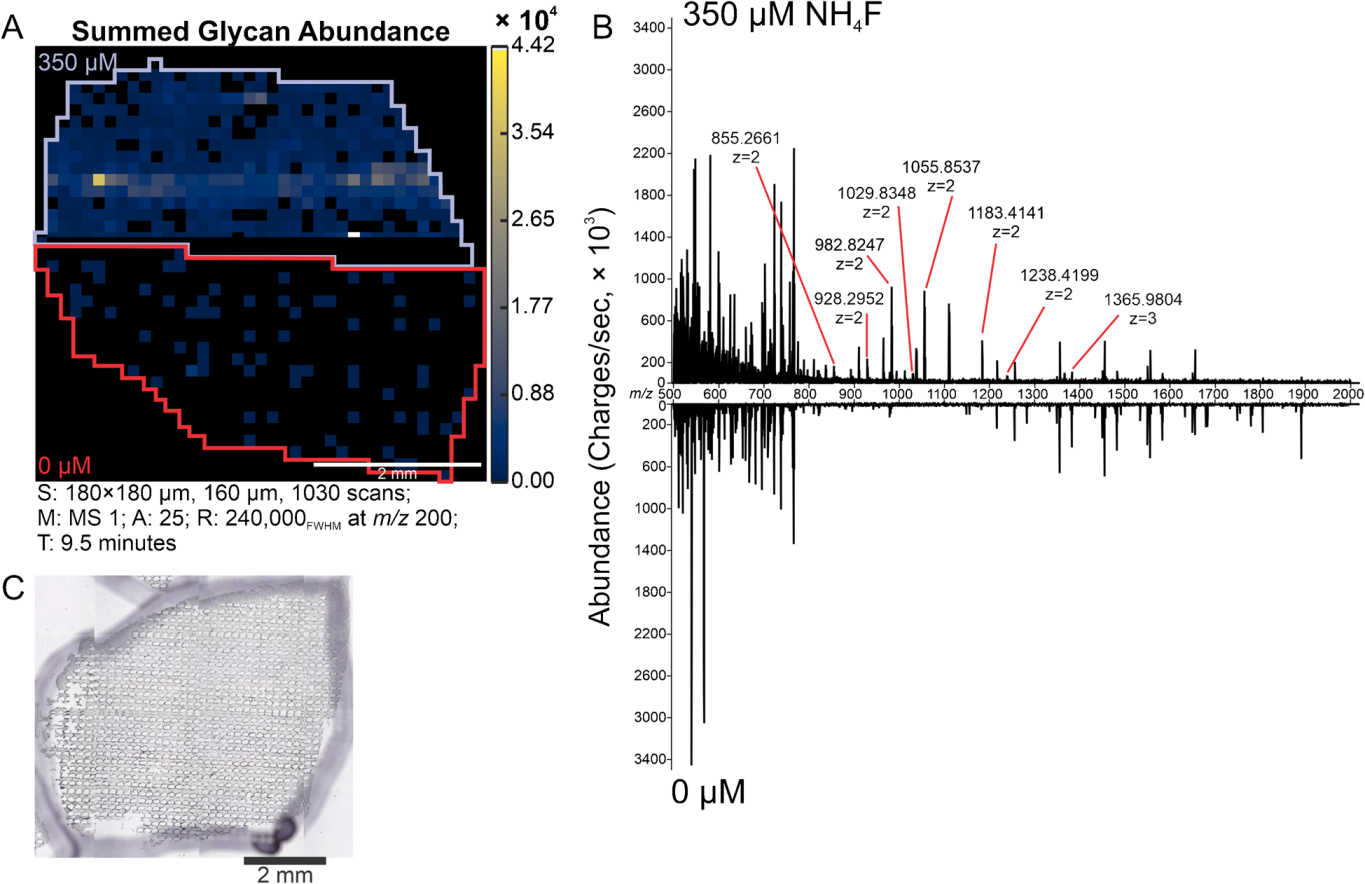
**A** FFPE human kidney tissue was imaged by IR-MALDESI with the top half at 0 µM NH_4_F and the bottom half at 350 µM. Summed abundance of 25 glycans are shown, unnormalized where the inclusion NH_4_F led to an increase in glycan signal, as well as the limit of detection, indicated by the significantly increased detection frequency (56% to 26%) of glycans across a relatively homogenous tissue. **B** Reflection plot showing representative mass spectra of undoped and NH_4_F doped signal across kidney tissue. Highly abundant glycans are indicated on the mass spectrum. **C** Optical image of the kidney post IR-MALDESI MSI imaging

To directly compare the signal enhancement caused by NH_4_F in tissue, kidneys were imaged without NH_4_F on the top half and with 350 µM NH_4_F on the bottom half (Fig. 5). The detected glycans (n = 25) match those previously detected using IR-MALDESI in human kidney, following the previously identified chloride adduction rule [28]. Both the detection frequency and abundance of glycans increased through the addition of the dopant.

**Fig. 6:**
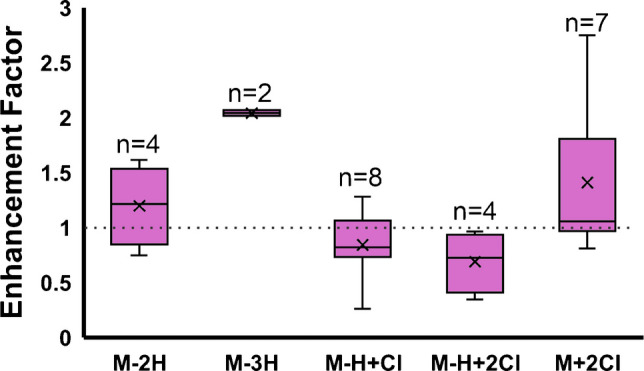
Box and whisker plot of 25 detected glycans showing the effect of charge state and adduct on glycan signal enhancement by ammonium fluoride. The enhancement factor was dependent on charge state and adduct type. Doubly- and triply-deprotonated glycans increased in signal. Chloride-adducted glycans that were also deprotonated showed a decrease in signal while glycans that were solely chloride-adducted had increased ion abundance with NH_4_F

In tissue imaging, a range of glycan adducts is observed, including [M-2H^+^]^2−^, [M-H^+^ + Cl^−^]^2−^, [M + 2Cl^−^]^2−^, [M-3H^+^]^3−^, and [M-2H^+^ + Cl^−^]^3−^. Chloride adducts are believed to be present from the glycan preparation process, which involves a citraconic acid buffer containing concentrated HCl [14]. The average signal enhancement of each type of adducted glycan is shown where a Kruskal-Wallis test showed statistically significant differences between the groups (Fig. 6).

Comparing the signal of glycans detected as [M-2H^+^]^2−^ and [M-3H^+^]^3−^ with and without the presence of NH_4_F, both showed enhanced ion abundance with the inclusion of NH_4_F in the ESI, of 1.2-fold and twofold, respectively (Fig. 6). This corresponds well with the proposed mechanism and indicates that F^−^ is abstracting protons from these molecules, similarly to lipids. The observed effect was larger for the triply deprotonated glycans, likely because these glycans have more labile protons that can be more easily deprotonated.

Conversely, glycans observed as [M-H^+^ + Cl^−^]^2−^ and [M-2H^+^ + Cl^−^]^3−^ showed decreased ion abundance with the presence of NH_4_F, decreasing between 1.2- and 1.4-fold. This is believed to be from a slight repulsion between the fluoride ion and the chloride adduct, preventing an interaction with the fluoride. Surprisingly, the [M + 2Cl^−^]^2−^ glycans were enhanced 1.4-fold, despite having no deprotonation events that could be affected by fluoride ions. We theorize that the presence of fluoride ions on the interior of the droplet further repels the chloride-adducted glycans, forcing them to the exterior of the droplet, resulting in increased desolvation and ionization, thereby increasing their signal [29, 30] .

## Conclusions

This work has demonstrated that the use of ammonium fluoride in negative mode IR-MALDESI leads to significant signal enhancement of lipids and glycans. The addition of 70 and 350 µM NH_4_F allows the F^−^ to capture protons from lipids and glycans, respectively, leading to the enhancement of [M-H^+^]^−^ ions. Biomolecules showed increased limits of detection and signal improvements of up to 93-fold, where lipids averaged eightfold and glycans showed up to a fourfold signal enhancement in solution and an average of 1.2-fold in tissue. Lipid signals were enhanced differently based on lipid class, whereas glycans showed increased signal with higher charge states and with a range of adducts.

## Supplementary Information

Below is the link to the electronic supplementary material.

Supplementary Material 1 (DOCX 312 KB)

## Data Availability

All putative lipid annotations by METASPACE are available: https://metaspace2020.org/project/ammonium_fluoride_MALDESI. All glycomics data is available upon request.
