# Supplementary material for: Signal enhancement of lipids and glycans using ammonium fluoride for IR-MALDESI mass spectrometry imaging
Source: Anal Bioanal Chem. 2025 Nov 18;417(30):7025–33. doi: 10.1007/s00216-025-06197-0 (PMC12680844; doi:10.1007/s00216-025-06197-0)
Supplement: Supplementary file 1 — Supplementary Material 1 (DOCX 312 KB) [file 216_2025_6197_MOESM1_ESM.docx]

**Supporting Information**

**Signal Enhancement of Lipids and Glycans Using Ammonium Fluoride for IR-MALDESI Mass Spectrometry Imaging**

Seth M. Eisenberg, Alora R. Dunnavant, Tana V. Palomino, and *David C. Muddiman

*Biological Imaging Laboratory for Disease and Exposure Research (BILDER), Department of Chemistry, North Carolina State University, Raleigh, NC 27695*

**Submitted to:** *Analytical and Bioanalytical Chemistry*

**Submitted**: October 13, 2025

**Supplemental Material**: 5 Pages / 2 Supplemental Figures / 3 Supplemental Tables

**Keywords**: IR-MALDESI; mass spectrometry imaging; electrospray doping; ammonium fluoride; glycans

***Author for Correspondence**

David C. Muddiman, Ph.D.

FTMS Laboratory for Human Health Research

Department of Chemistry

North Carolina State University

Phone: 919-513-0084

Email: [dcmuddim@ncsu.edu](mailto:dcmuddim@ncsu.edu)

**Supplemental Table 1.** Annotation list for lipids included in graph and heatmap in Figure 2. The identifications are provided by METASPACE with 10% FDR. The METASPACE datasets are available at <https://metaspace2020.org/api_auth/review?prj=2830b3b2-5cf2-11f0-a049-bbe54258a833&token=BeyTsOuNxuNL>.

| *m/z* | Formula | Putative Identification |
| --- | --- | --- |
| 158.0823 | C7H13NO3 | FA |
| 161.0455 | C6H10O5 | FA 6:1; O3 |
| 175.0248 | C6H8O6 | FA 6:2; O4 |
| 179.0561 | C6H12O6 | FA 6:0; O4 |
| 202.1085 | C9H17NO4 | CAR 2:0 |
| 277.2173 | C18H30O2 | FA 18:3 |
| 279.233 | C18H32O2 | FA 18:2 |
| 281.2486 | C18H34O2 | FA 18:1 |
| 283.2643 | C18H36O2 | FA 18:0 |
| 301.2173 | C20H30O2 | PR |
| 301.2384 | C17H34O4 | FA 17:0; O2 |
| 303.233 | C20H32O2 | FA 20:4 |
| 305.2486 | C20H34O2 | FA 20:3 |
| 307.2643 | C20H36O2 | FA 20:2 |
| 309.2799 | C20H38O2 | FA 20:1 |
| 325.2384 | C19H34O4 | FA 19:2; O2 |
| 327.233 | C22H32O2 | FA 22:6 |
| 327.2541 | C19H36O4 | FOH 19:2; O3 |
| 329.2486 | C22H34O2 | FA 22:5 |
| 329.2697 | C19H38O4 | MG 16:0 |
| 331.2643 | C22H36O2 | FA 22:4 |
| 379.0823 | C21H16O7 | PK |
| 391.2255 | C19H37O6P | CPA 16:0 |
| 393.2646 | C23H38O5 | ST 23:1; O5 |
| 401.2909 | C22H42O6 | WE 22:1; O4 |
| 417.2411 | C21H39O6P | CPA 18:1 |
| 419.2568 | C21H41O6P | CPA 18:0 |
| 435.2517 | C21H41O7P | PA 18:1 |
| 599.3202 | C27H53O12P | PA 18:0 |
| 606.0743 | C17H27N3O17P2 | SL |
| 671.4657 | C37H69O8P | PA 34:2 |
| 673.4814 | C37H71O8P | PA 34:1 |
| 695.4657 | C39H69O8P | PA 36:4 |
| 697.4814 | C39H71O8P | PA 36:3 |
| 699.497 | C39H73O8P | PA 36:2 |
| 719.4657 | C41H69O8P | PA 38:6 |
| 721.4814 | C41H71O8P | PA 38:5 |
| 723.497 | C41H73O8P | PA 38:4 |
| 725.5127 | C41H75O8P | PA 38:3 |
| 738.5079 | C41H74NO8P | PC 33:4 or PE 36:4 |
| 745.4814 | C43H71O8P | PA 40:7 |
| 747.497 | C43H73O8P | PA 40:6 |
| 747.5182 | C40H77O10P | PG 34:1 |
| 749.5127 | C43H75O8P | PA 40:5 |
| 762.5079 | C43H74NO8P | PC 35:6 or PE 38:6 |
| 764.5236 | C43H76NO8P | PC 35:5 or PE 38:5 |
| 766.5392 | C43H78NO8P | PC 35:4 or PE 38:4 |
| 773.5127 | C45H75O8P | PA 42:7 |
| 790.5392 | C45H78NO8P | PC 37:6 or PE 40:6 |
| 834.5291 | C46H78NO10P | PS 40:6 |
| 885.5499 | C47H83O13P | PI 38:4 |


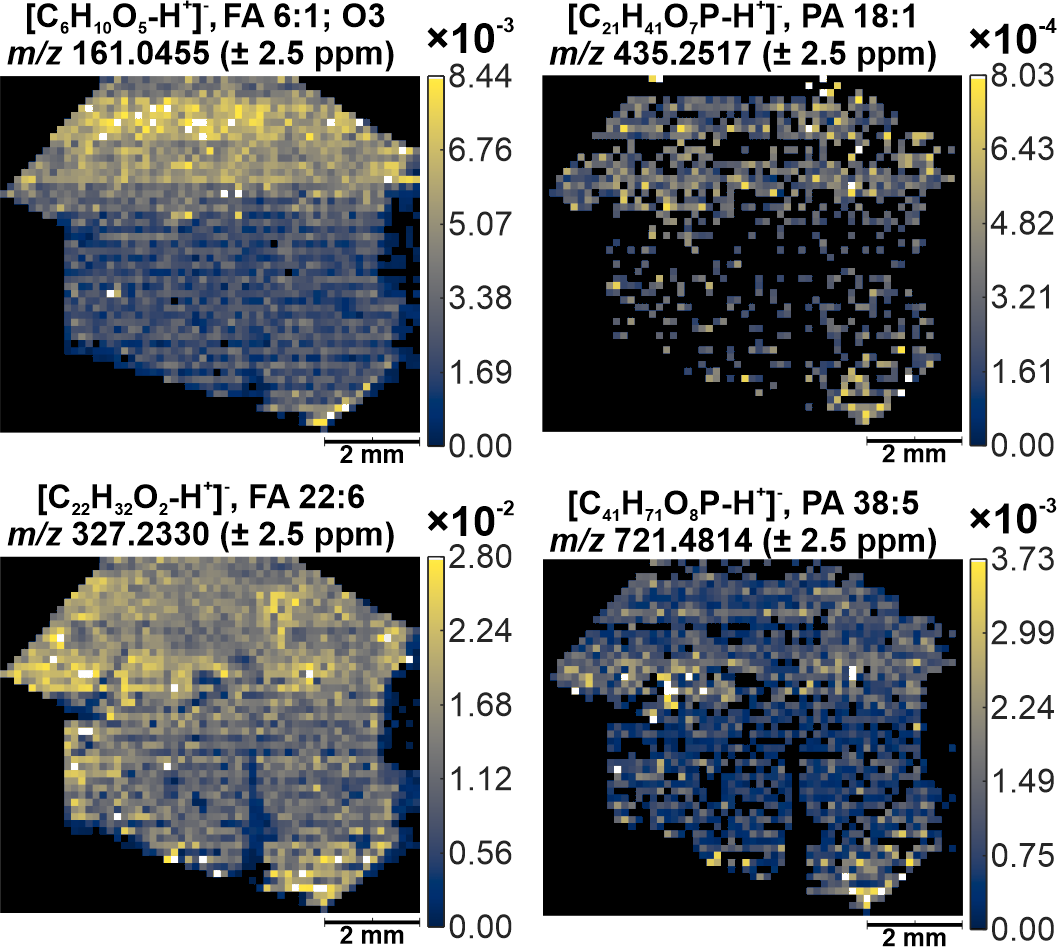


**Supplemental Figure 1.** TIC normalized heatmaps for selected *m/z* from annotated lipids shown in Table 1. These lipids show increased abundance near the top of the tissue, aligning with the 60-80 µM NH_4_F range. As the concentration of NH_4_F increased, the signal enhancement decreased.


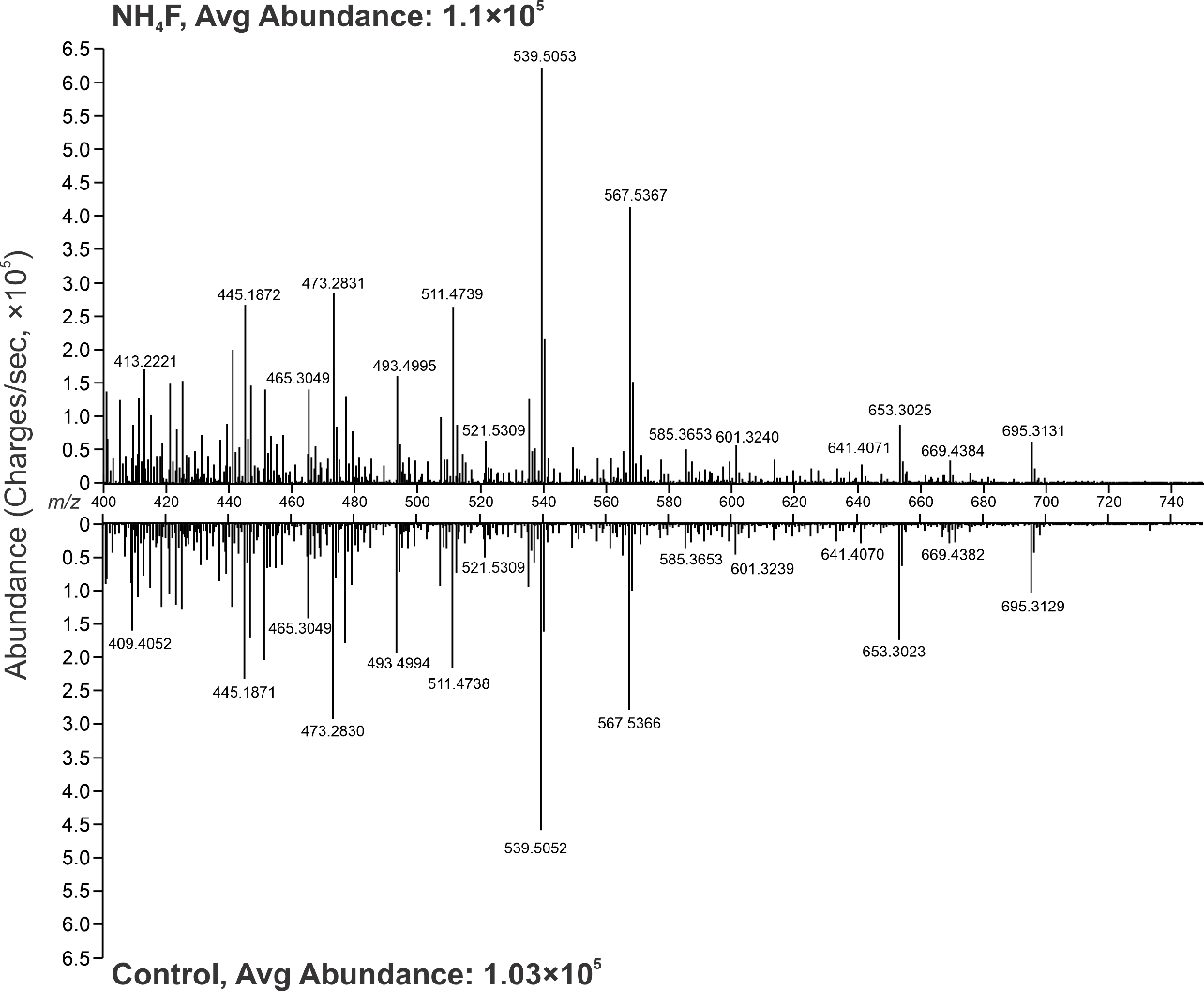


**Supplemental Figure 2.** Reflection plot mass spectrum comparing a background spectrum from NH4F-doped ESI (top) and control ESI (bottom). Very few differences are observed between the spectra and the average abundances across the biologically relevant range show no significant differences (1.1E5 vs. 1.03E5).

**Supplemental Table 2.** All p-values for comparisons between lipid classes. P-values calculated using a Nemenyi test with a Tukey correction in R.

|  | FA | LPA | LPE | LPG | LPI | PA | PE | PG |
| --- | --- | --- | --- | --- | --- | --- | --- | --- |
| LPA | 0.96055 | - | - | - | - | - | - | - |
| LPE | 0.9998 | 0.97666 | - | - | - | - | - | - |
| LPG | 0.36005 | 0.81253 | 0.41989 | - | - | - | - | - |
| LPI | 0.99471 | 0.87329 | 1.00000 | 0.26976 | - | - | - | - |
| PA | 0.00178 | 0.01050 | 0.85134 | 0.01434 | 0.97634 | - | - | - |
| PE | 7.0e-14 | 1.3e-1 | 9.7e-5 | 5.9e-7 | 0.00060 | 1.3e-5 | - | - |
| PG | 0.00156 | 0.00228 | 0.43050 | 0.00332 | 0.69255 | 0.95641 | 0.05870 | - |
| PI | 1.9e-7 | 3.2e-7 | 0.00095 | 3.2e-6 | 0.00337 | 0.00367 | 0.99962 | 0.13215 |

**Supplemental Table 3.** Putative identification and *m/z* of 14 lipids with an enhancement factor of greater than 20-fold, indicating it was uniquely observed while using NH_4_F ESI.

| *m/z* | Formula | Putative Identification |
| --- | --- | --- |
| 235.1704 | C_15_H_24_O_2_ | PR |
| 275.2017 | C_18_H_28_O_2_ | FA 18:4 |
| 311.2956 | C_20_H_40_O_2_ | FA 20:0 |
| 395.2803 | C_23_H_40_O_5_ | FA 23:3; O3 |
| 399.2541 | C_25_H_36_O_4_ | PR |
| 423.2752 | C_23_H_20_O_8_ | PK |
| 437.2909 | C_21_H_43_O_7_P | LPA 18:0 |
| 443.2568 | C_23_H_41_O_6_P | LPA O-20:4 |
| 461.2909 | C_27_H_42_O_6_ | ST 27:3; O6 |
| 498.2895 | C_26_H_45_NO_6_S | ST 24:1; O3; Tau |
| 505.2572 | C_24_H_43_O_9_P | LPG 18:3 |
| 509.4575 | C_32_H_62_O_4_ | FA 32:1; O2 |
| 512.2687 | C_26_H_43_NO_7_S | ST |
| 514.2844 | C_26_H_45_NO_7_S | ST 24:1; O4; Tau |
| 587.5045 | C_38_H_68_O_4_ | FA 36:3; O |
